# 

**Published:** 1912-12

**Authors:** 


					TOau-
CONTENTS. page
?Robert
ffletcber.
By Sir William Osler, Bart., M.D., F.R.S.
289
On ?
iu Diseases Bearing Names of Saints.
AVI H  ??_ j T"? *_._l n/r t-> r* o
By Robert Fletcher,
Columbia and Bristol, M.R.C.S.
TL
295
^^?"oblem of Medi cat Education.
__ i t-> ? ?
By Walter C. Swaynk, M.D.
T ~ wiii v/| |VICU
ond. and Bristol
.uuuauun. xjy y* Ai-ir.iv v^. ivx.ju/.
3*6
Barber Surgeons.
By George Parker, M.D., M.A. Cantab.
327
Y ? oww,m"
^ ^er?u''n and Immunity.
By John Wm. Taylor, M.D. Bristol
338
T"he
Bristol Medical Library.
By C. King Rudge, M.R.C.S. Eng.,
T iV' mcuiUdl
L ^C.P., Lond.
344
lr  > ^uuu-  J44
?^ress of the Medical Sciences:
Medic
"cine.
?...
By J. M. Fortescue-Brickdale, M.A., M.D., B.Ch.Oxon.
35?
^Urgery.
C. Ferrier Walters, F.R.C.S.
354
views of Books
359
Frl-. *-><-><
No'l?:iai No,?
367
^otes
>, ?n Preparations for the Sick
... 369
? ? 1 cparaxions tot xne oick  309
library of the Bristol Medico-Chirurgical Society .../ ... 373
? y wI me d
*'ngs of Societies
r>i_.
Hl. ?
. ,tUapy Notices
... ... *>?>&. 382
i '     ^n\382
Cal Medical Notes  jQ \9*1'3 '"Jjj383

				

